# The circular RNA expression profile in ovarian serous cystadenocarcinoma reveals a complex circRNA–miRNA regulatory network

**DOI:** 10.1186/s12920-021-01132-5

**Published:** 2021-12-02

**Authors:** Minhui Zhuang, Jian Zhao, Jing Wu, Shilong Fu, Ping Han, Xiaofeng Song

**Affiliations:** 1 Department of Biomedical Engineering, Nanjing University of Aeronautics and Astronautics, Nanjing, 211106 China; 2 School of Biomedical Engineering and Informatics, Nanjing Medical University, Nanjing, 211166 China; 3 Department of Gynecology and Obstetrics, The First Affiliated Hospital With Nanjing Medical University, Nanjing, 210029 China

**Keywords:** circRNA, Expression profile, Interaction network, Ovarian serous cystadenocarcinoma

## Abstract

**Background:**

Ovarian serous cystadenocarcinoma is one of the most serious gynecological malignancies. Circular RNA (circRNA) is a type of noncoding RNA with a covalently closed continuous loop structure. Abnormal circRNA expression might be associated with tumorigenesis because of its complex biological mechanisms by, for example, functioning as a microRNA (miRNA) sponge. However, the circRNA expression profile in ovarian serous cystadenocarcinoma and their associations with other RNAs have not yet been characterized. The main purpose of this study was to reveal the circRNA expression profile in ovarian serous cystadenocarcinoma.

**Methods:**

We collected six specimens from three patients with ovarian serous cystadenocarcinoma and adjacent normal tissues. After RNA sequencing, we analyzed the expression of circRNAs with relevant mRNAs and miRNAs to characterize potential function.

**Results:**

15,092 unique circRNAs were identified in six specimens. Approximately 46% of these circRNAs were not recorded in public databases. We then reported 353 differentially expressed circRNAs with oncogenes and tumor-suppressor genes. Furthermore, a conjoint analysis with relevant mRNAs revealed consistent changes between circRNAs and their homologous mRNAs. Overall, construction of a circRNA–miRNA network suggested that 4 special circRNAs could be used as potential biomarkers.

**Conclusions:**

Our study revealed the circRNA expression profile in the tissues of patients with ovarian serous cystadenocarcinoma. The differential expression of circRNAs was thought to be associated with ovarian serous cystadenocarcinoma in the enrichment analysis, and co-expression analysis with relevant mRNAs and miRNAs illustrated the latent regulatory network. We also constructed a complex circRNA–miRNA interaction network and then demonstrated the potential function of certain circRNAs to aid future diagnosis and treatment.

**Supplementary Information:**

The online version contains supplementary material available at 10.1186/s12920-021-01132-5.

## Background

Ovarian cancer is a common type of tumor formed in the female reproductive system that poses a serious threat to the health of women [1]. Ovarian serous cystadenocarcinoma is an epithelial subtype of ovarian cancer that is difficult to cure. Because of the lack of effective screening technology and inconspicuous early-stage symptoms, diagnosis is often not made until advanced stages [2]. There is thus a need to develop more accurate methods for early detection. Researchers have already noticed that some features at the molecular level might provide signs of underlying illness. For example, family history was thought to indicate risk for ovarian cancer, which might be caused by genomic mutations [3]. Moreover, dysregulation of special genes associated with adverse prognosis in ovarian cancer, such as p53 and VEGF, suggested that they have the potential to be used as biomarkers in diagnosis and treatment [4, 5]. However, the molecular mechanisms underlying ovarian serous cystadenocarcinoma remain unclear.

Circular RNA (circRNA) is a type of noncoding RNA with a special molecular structure. Although they were first discovered from RNA viruses over forty years ago, circRNAs were not confirmed to exist in human cells until 1991 [6–8]. circRNAs are different from their homologous linear transcripts in splicing forms, as they are typically formed by the back-splicing of exons from precursor mRNA (pre-mRNA). This procedure permits circRNAs to exist as RNA circles without polyadenylated (polyA) tails. In some cases, circRNAs can be relatively long-lived and weakly expressed [9]. In other cases, circRNAs can accumulate when they were abundant at a steady-state level post-transcriptionally [10]. Researchers have also recently found that the abnormal expression of circRNAs might be involved in human diseases. The abundance of circRNAs in colorectal cancer cell lines has been reported to be globally reduced, and a negative correlation between global circRNA abundance and proliferation has been validated for several diseases, including ovarian cancer [11]. In addition, some studies have suggested that circRNAs provide important indicators with prognostic implications. Hsa_circ_0001649 has been reported to be downregulated and associated with tumor size and differentiation grade in cholangiocarcinoma, indicating its potential as a cancer-related therapeutic target [12]. Another circRNA cSMARCA5 was suggested to provide a potential therapeutic target of hepatocellular carcinoma because it could mediate TIMO3 expression and inhibit the migration and proliferation of hepatocellular carcinoma cells [13]. In ovarian cancer, Ning et al. found that circEXOC6B and circN4BP2L2 can act as potential prognostic biomarkers [14]. Although several studies of circRNAs have been conducted, there is still a need for additional research to explore the mechanisms underlying human diseases.

The function of circRNAs in tumorigenesis might be associated with their complex biological mechanisms. At the expression level, circRNAs have been found to compete with linear splicing and act as regulators [15]. Another remarkable function of circRNAs is their ability to participate in regulation as microRNA (miRNA) sponges. For example, ciRS-7/CDR1as (CDR1 antisense) is often noted to function as a sponge of miR-7. This single-exon circularized RNA was determined to contain over 60 selectively conserved miRNA binding sites [16, 17] that allow it to function as a negative regulator for miR-7 through competitive combination and then modulate the expression of oncogenes targeted by mir-7 [18]. Moreover, testis-specific circRNA Sry (sex-determining region Y) has been observed to function as a sponge of miR-138, which could inhibit cell migration and invasion in ovarian and breast cancer cells by targeting SOX4 and HIF-1α [15, 19]. Therefore, the regulatory network related to circRNAs may help clarify tumorigenesis in ovarian serous cystadenocarcinoma.

Here, a transcriptome analysis was conducted to characterize the function of circRNAs associated with ovarian serous cystadenocarcinoma. We detected the differential expression of circRNAs in several tissues. Furthermore, a co-expression analysis with relevant mRNAs and miRNAs revealed several special circRNAs that could serve as potential biomarkers.

## Methods

### Specimen collection and RNA sequencing

We collected six specimens from three patients with ovarian serous cystadenocarcinoma. Three tumor specimens came from tumor tissues, and the other three normal specimens came from para-tumor tissues. All patients were informed of the purpose of the study and signed informed consents; the collection of specimens was approved by the ethics committee of Jiangsu Province Hospital. Total RNA from each specimen was extracted using the RNAsimple total RNA kit (Tiangen, China) per the manufacturer's instructions. cDNA fragments of 250–300 bp were isolated and then subjected to approximately 20 cycles of PCR amplification. For end repair and joint connection, we used the Illumina TruSeq total RNA library preparation kit. After further purification and quality detection of the amplified products, the total RNA library preparation was completed. Transcriptome sequencing was performed using HiSeq4000 (Illumina).

### Identification of human circRNAs and mRNAs

To identify circRNAs and mRNAs, several pipelines were used. We used BWA to build the index of the reference genome and BWA-MEM, and the alignment algorithm was implemented as a component of BWA to map the paired-end reads of each specimen to the human reference genome [20]. The FASTA-formatted sequence file of the human reference genome was downloaded from the UCSC Genome Browser (GRCh37/hg19) [21]. We then used CIRI2 to identify circRNAs in tissues [22, 23]. Based on multiple seed matching, CIRI2 can use an adapted maximum likelihood estimation to identify back-spliced junction reads. Based on the mRNAs in tissues, the expression level was evaluated with Tophat2 and Cufflinks [24, 25]. Cufflinks can assemble transcripts and evaluate their abundances from aligned RNA-Seq reads after mapping by Tophat2.

### Dataset for bioinformatic analysis

Data on known circRNAs were downloaded from circBase (H.sapiens updated in July 2017) [26]. For the co-expression analysis with miRNAs, experimentally validated tumorigenesis miRNAs were collected from miR2Disease (http://www.mir2disease.org/) together with their references [27]. Meanwhile, human miRNA–target interactions (MTIs) were directly downloaded from miRTarBase Release 7.0 (http://mirtarbase.mbc.nctu.edu.tw/) [28]. miRTarBase provides several validated MTIs from collected research articles related to functional studies of miRNAs. Furthermore, genes validated from reporter assays are generally thought to provide strong evidence for interaction with miRNAs. In the end, data on human oncogenes and tumor-suppressor genes were collected from two public databases: ONGene and TSGene 2.0 [29, 30]

### Functional enrichment analysis

To conduct the functional enrichment analysis, we used Metascape, which is a set of reliable and intuitive tools that can obtain gene annotations and can be used to conduct gene list enrichment analyses (last update date: 2018-11-20) [31]. Metascape integrates a few related databases and provides a more comprehensive and updated enrichment analysis for gene lists. Standard accumulative hypergeometric statistical tests were used, and results were ordered by their p-values.

### Construction of a circRNA-miRNA network

In the co-expression analysis with related miRNAs, the miRanda pipeline was used to predict the interactions between miRNAs and circRNAs [32]. MiRanda provides a miRNA target scanner using dynamic-programming alignments and thermodynamics for assessments. Furthermore, miRNAs experimentally validated in connection with ovarian cancer were used to construct the circRNA-miRNA network.

## Results

### Landscape of circRNAs in ovarian serous cystadenocarcinoma

**Fig. 1:**
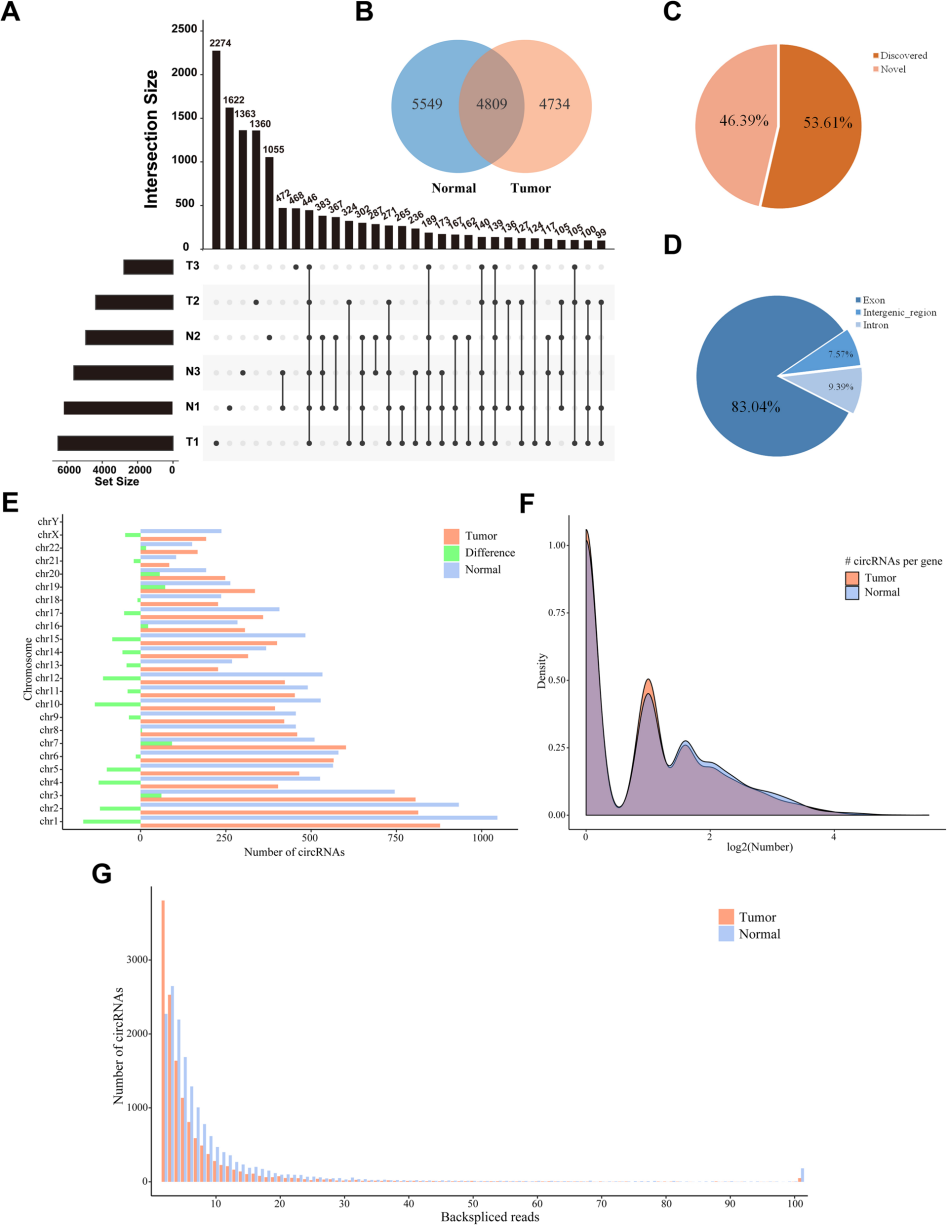
**A** Intersections of identified circRNAs in six specimens. **B** Overlap of identified circRNAs in tumor and normal groups. **C** Distribution of circRNAs with their annotations. **D** Distribution of circRNAs according to formation type. **E** Distribution of circRNAs on 23 human chromosomes (without chrY in ovarian tissues). Difference = number of circRNAs in tumor tissues—number of circRNAs in normal tissues. **F** Comparison of isoform number per gene between tumor and normal groups. **G** Distribution of circRNAs ordered by the number of back-spliced reads

Overall, we found 15,092 unique circRNAs with at least 2 junction reads spanning a head-to-tail splice junction. A total of 6500, 4370, and 2774 circRNAs were identified in the three tumor specimens, and 6201, 4932, and 5606 circRNAs were found in the three normal specimens (Fig. 1A). And there was no correlation between sequence depths and numbers of detection (Additional file 1: Fig. S1). A total of 446 unique circRNAs appeared in the six specimens. Overall, 9543 unique circRNAs in tumor tissues and 10,358 in normal tissues shared the intersection composed of 4809 individuals (Fig. 1B). Furthermore, the proportion of novel individuals indicated that the circRNAs identified in these specimens were worth studying (Fig. 1C). We searched the identified circRNAs using circBase and found that nearly half of these circRNAs were not recorded in this database. We then calculated the proportion of different formation types of circRNAs. As expected, the majority of circRNAs (approximately 83%) were annotated with the type of exon (Fig. 1D).

Given the above results, we further explored the abundance of circRNAs in different tissues. The distribution of circRNAs on human chromosomes is shown as bar charts (Fig. 1E). All circRNAs were distributed on each chromosome separately (except allosome Y); the differences were plotted in green bars for clarity. Despite the fact that there were more circRNAs in tumor tissues on some chromosomes, we still noticed that the numbers of circRNAs per chromosome in normal tissues were generally larger. Meanwhile, circRNAs were found to be especially enriched on the first three chromosomes. A possible reason for this observation was that the first three autosomes were relatively long. We then calculated the numbers of circRNA isoforms per gene (Fig. 1F). Overall, the normal tissues tended to have more homologous circular transcripts, although the gene PATK2 in tumor tissues had the most (46). The range was from 0 to 5, and most circRNAs in tumor tissues tended to have one or no homologous isoform. We also determined the distribution based on the calculated numbers of back-spliced reads (with number = 1 ignored). The number of circRNAs in the tumor group was unexpectedly larger than the other when the number of back-spliced reads was 2 (Fig. 1G). Despite the fact that the total number of circRNAs in normal tissues was larger, tumor tissues tended to amplify the low expression level of some circRNAs. As mentioned above, the expression patterns of circRNAs were clearly different between tumors and normal tissues.

### Differential expression of circRNAs in human tissues

**Fig. 2:**
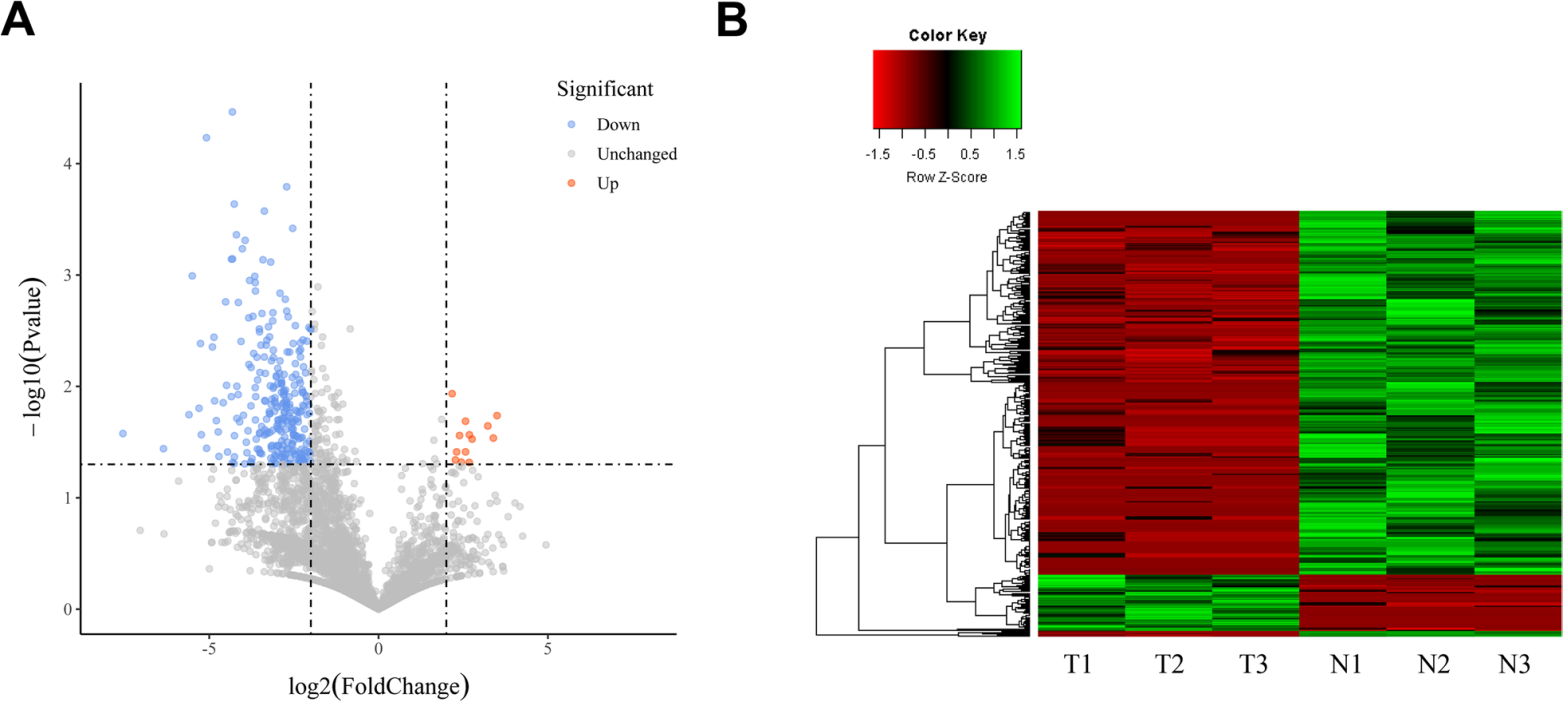
**A** Volcano plots demonstrated statistical differences in circRNA expression. Red dots represent the upregulated circRNAs in tumor tissues. **B** Heatmaps showed the differential expression profile of 353 selected circRNAs in tissues

To further assess the function of circRNAs in human tissues, we performed differential expression analysis. Quantification of circRNAs was conducted using the number of back-spliced reads and then was normalized by RPM (reads per million mapped reads). In terms of expression, more circRNAs were downregulated in tumor tissues. Categorization was performed using a volcano plot (Fig. 2A). Moreover, data obtained after the stability assessment were used to examine differences between tissues. Some of the circRNAs were only significantly expressed in all three specimens of the tumor group or normal group. These group-specific species could also explain the function of circRNA. To highlight the differences, the expression level of the selected circRNAs mentioned above are shown in the heatmap (Fig. 2B).

**Fig. 3:**
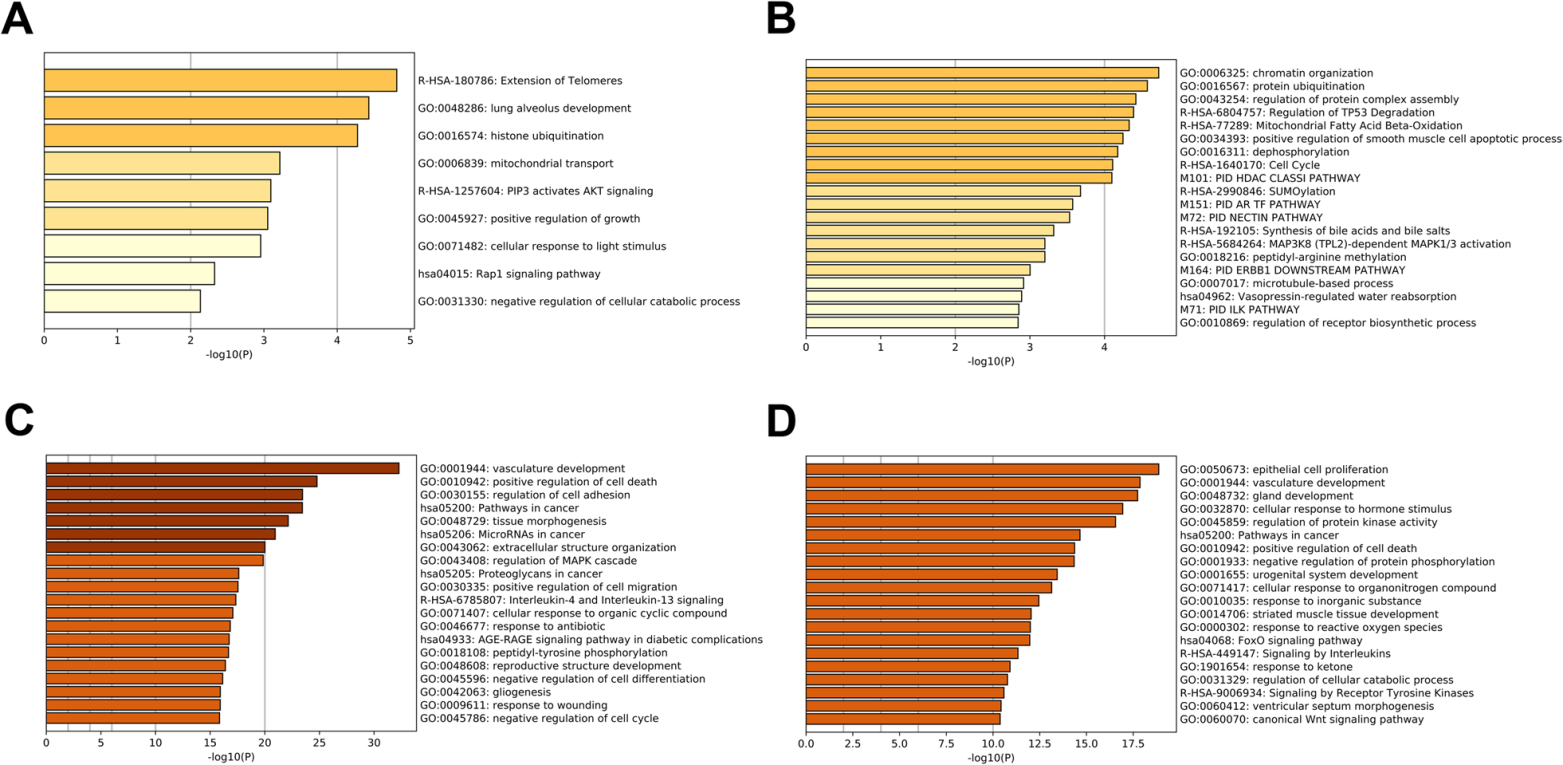
**A** Representative enriched terms for circRNAs upregulated in tumor tissues. **B** Representative enriched terms for circRNAs downregulated in tumor tissues. **C** Representative enriched terms for miRNA-target genes upregulated in tumor tissues. **D** Representative enriched terms for miRNA-target genes downregulated in tumor tissues

**Fig. 4:**
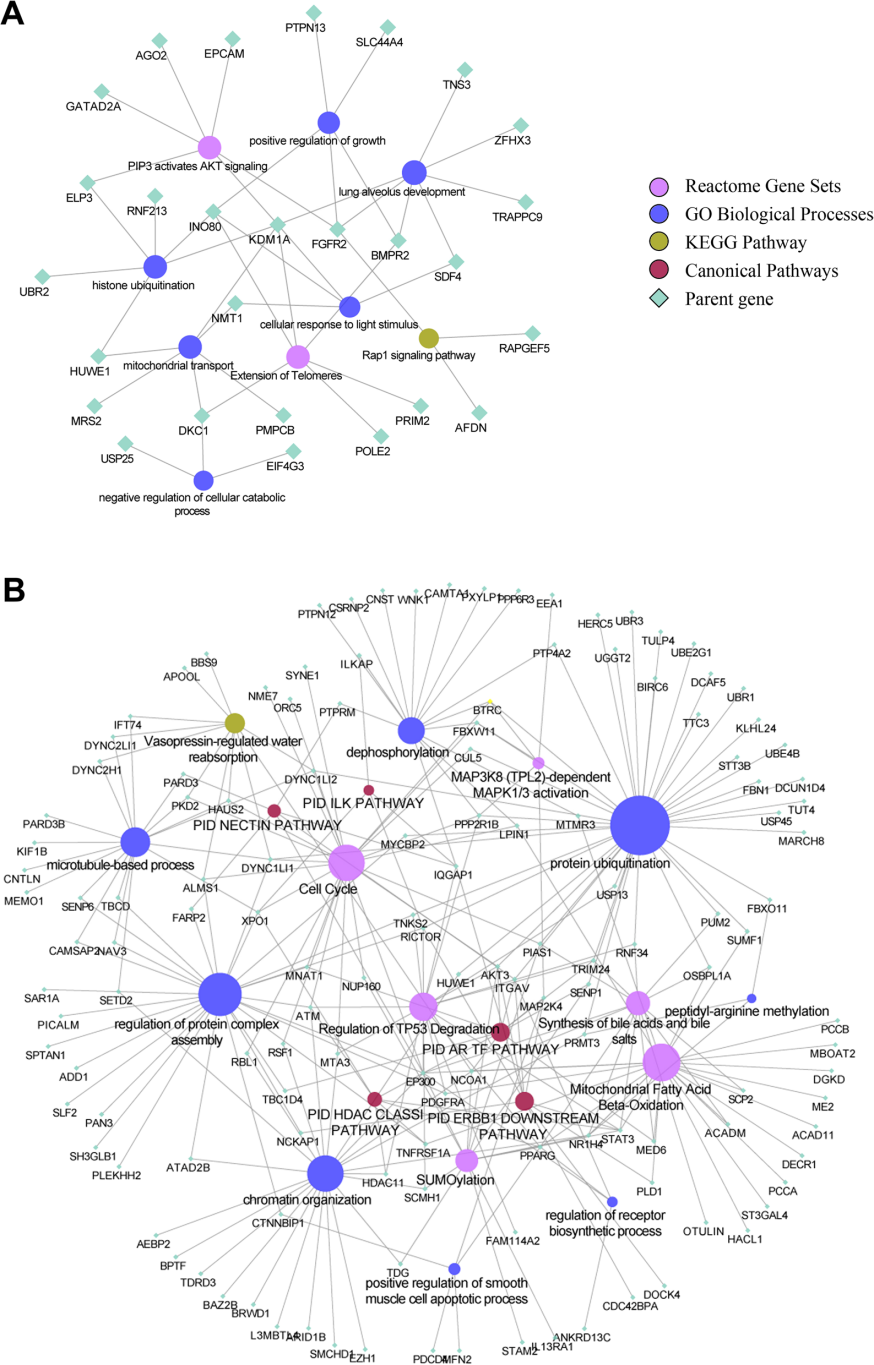
The network produced by the functional enrichment analysis. The colors and shapes represented different categories, and the size of circular nodes represented the counts of genes for each term. **A** Pathway network of circRNAs upregulated in tumor tissues. **B** Pathway network of circRNAs downregulated in tumor tissues

Based on the screening, enrichment analysis was conducted with Metascape. Parent genes of circRNAs were used to conduct separate searches for relevant functions. Because of the disparity between the numbers in the two groups, the results of the enrichment analysis also revealed a major difference in the number of terms. Representative enriched terms of the top 9 clusters in the upregulated group tended to be GO biological processes focused on growth and regulation (Fig. 3A). GO:0031330: negative regulation of cellular catabolic process and GO:0045927: positive regulation of growth indicated that these circRNAs were possibly related to the abnormal growth and degradation of tumor cells. In addition, R-HSA-1257604: PIP3 activates AKT signaling had slightly more counts in the enriched terms. This pathway might reflect a more direct relationship to ovarian serous cystadenocarcinoma. Over the past few years, several studies have explored the connection between AKT/pAKT and the invasion and metastasis of ovarian cancer [33]. In a study of FSH-stimulated VEGF expression in ovarian serous cystadenocarcinoma, the activation of the PI3K/AKT pathway mediated the upregulated expression of survivin and then promoted VEGF expression [34]. Moreover, positive AKT and phosphorylated AKT (pAKT) protein expression was associated with poor prognosis. Representative enriched terms of the top 20 clusters in the downregulated group tended to be related to the maintenance of life activities (Fig. 3B). We observed that some enriched terms were related to the normal functions of the human body, such as chromatin organization and protein ubiquitination. The canonical pathway, M164: PID ERBB1 downstream pathway, suggested that para-tumor tissues were likely fighting tumor invasion. A previous study demonstrated that the inhibition of ERBB1 and ERBB2 could rapidly inhibit the motility and intravasation of tumor cells [35]. In such cases, circRNAs of para-tumor tissues have been suggested to play a role in self-regulation for defense. We then constructed a network for enrichment analysis. From this network, we observed that some genes were involved in several terms. Among the top 9 clusters in the upregulated group, KDM1A (also known as LSD1) appeared in 6 terms (Fig. 4A). Among the top 20 representative enriched terms in the downregulated group, EP300 was associated with 11 terms (Fig. 4B). In previous studies, the expression of LSD1 was found to gradually increase from ovarian benign cystadenoma and borderline cystadenoma to cystadenocarcinoma [36]. Likewise, the overexpression of LSD1 mRNA has been observed in human ovarian tumors [37]. Given that the circRNA of KDM1A was also overexpressed in tumor tissues, this gene might be a potential focus for future study.

**Fig. 5:**
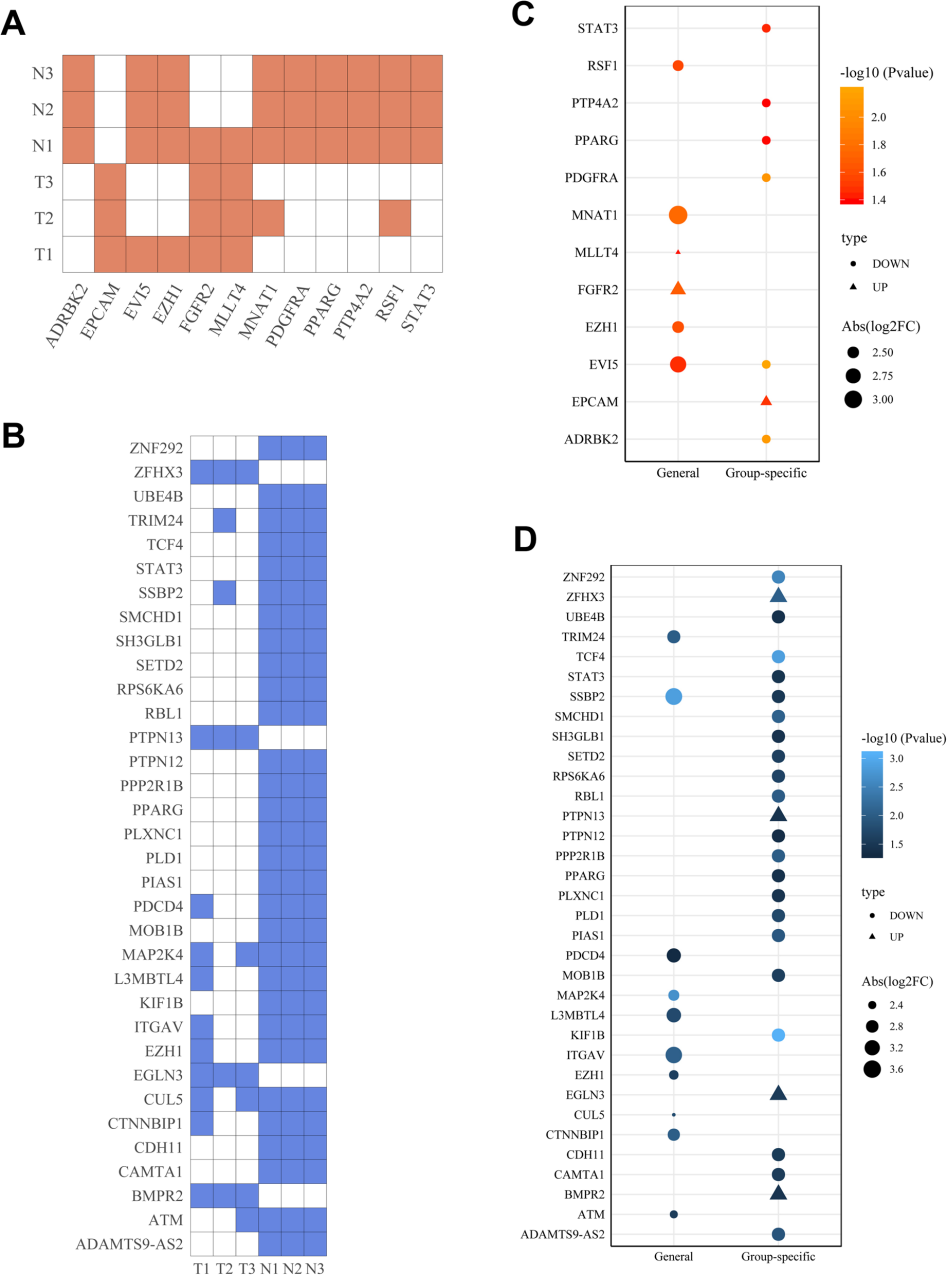
**A** Occurrence of oncogenes in differentially expressed circRNAs. **B** Occurrence of tumor-suppressor genes in differentially expressed circRNAs. **C** Distribution of oncogenes in differentially expressed circRNAs in tissues. "Group-specific" meant that some differentially expressed circRNAs were only significantly expressed in all three specimens of one group and were not expressed in the other group. **D** Distribution of tumor-suppressor genes in differentially expressed circRNAs in different tissues

To explore the potential function of circRNAs in tumor progression, we conducted a conjoint analysis with oncogenes and tumor-suppressor genes. Among the selected circRNAs, we found that 12 parental genes of 14 circRNAs were annotated as oncogenes (Fig. 5A). We also identified 34 tumor-suppressor genes that had circular transcripts (Fig. 5B). We also found that group-specific circRNAs accounted for the majority of the circRNAs (Fig. 5C, D). This finding further suggested that particular species functioned in tumor progression. For example, the oncogene EPCAM, which had two novel circular isoforms, was only expressed in tumor tissues and is known to contribute to the formation of the protein epithelial cellular adhesion molecule (EpCAM). The human EpCAM protein has been shown to be highly expressed in many samples of human cancer (including ovarian cancer) and is considered a potential immunotherapeutic target for treatment [38].

### Co-expression analysis with mRNAs and miRNAs

**Fig. 6:**
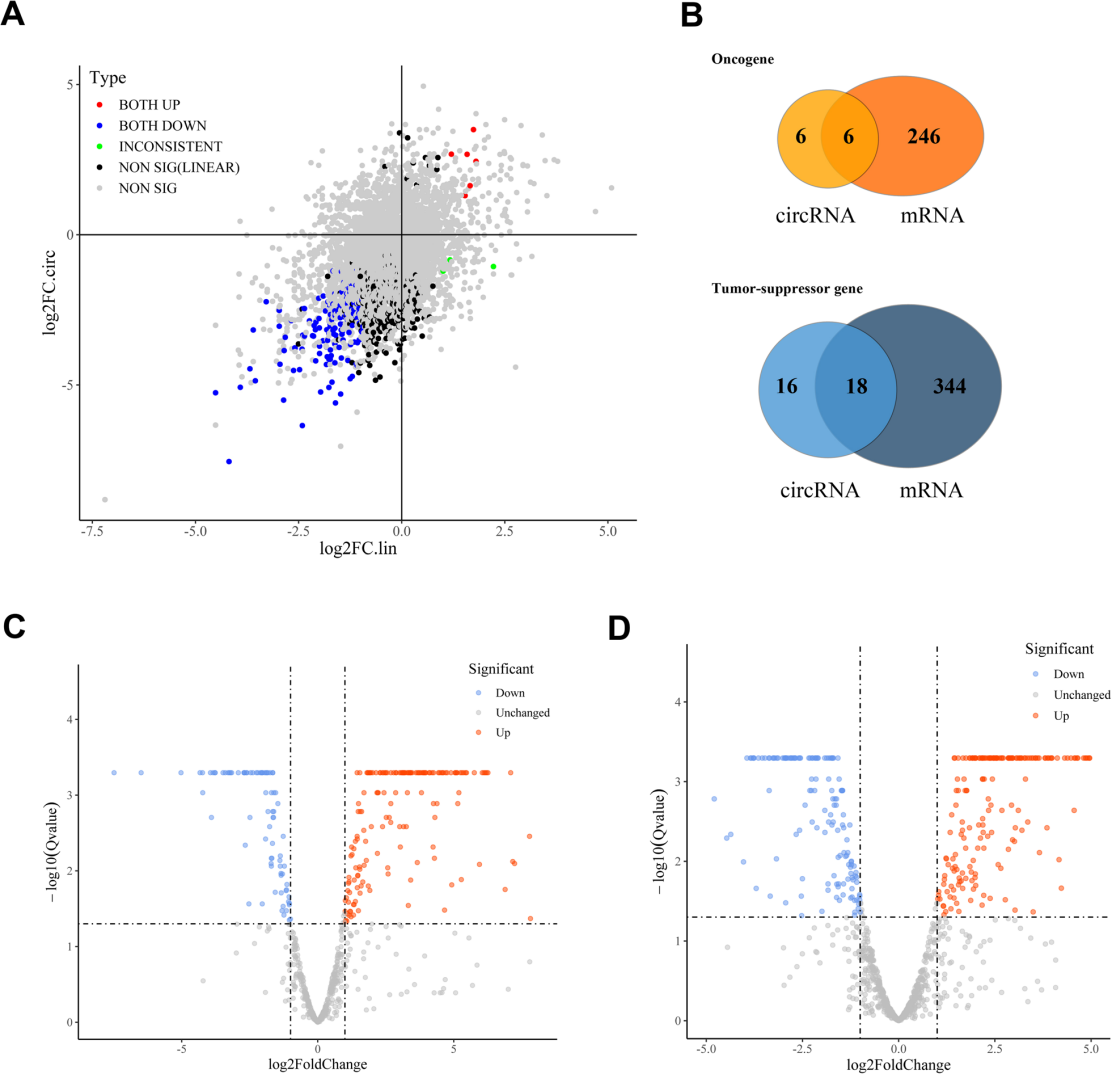
**A** The correlation between the expression of circRNAs and their homologous mRNAs measured by log2Foldchange. The red and blue points indicated that circRNAs and their linear transcripts were either significantly upregulated or downregulated in tumor tissues, respectively. Green points indicated that circRNAs and their linear transcripts had different patterns of change. However, some circRNAs (black points) were expressed differentially when their respective mRNAs did not show parallel changes. **B** Overlap in the occurrence of oncogenes and tumor-suppressor genes between differentially expressed circRNAs and mRNAs. **C** Distribution of oncogenes in differentially expressed mRNAs in tissues. **D** Distribution of tumor-suppressor genes in differentially expressed mRNAs in tissues

Next, we conducted a co-expression analysis with mRNAs and miRNAs to establish the potential relevance. First, we compared the expression patterns of circRNAs and their homologous mRNAs (Fig. 6A), and expression abundance appeared important. We found that the majority of circRNAs and their respective linear transcripts whose expression significantly changed tended to be downregulated in tumor tissues. Only a few of them showed the opposite pattern of expression. However, some circRNAs exhibited marked changes in their expression level, but the expression level of their mRNAs did not significantly vary. A similar pattern was observed for a portion of the circRNAs that could not be shown in the scatter diagram. We then evaluated the performance of the linear transcripts of oncogenes and tumor-suppressor genes (Fig. 6C, D). And there was overlap among approximately half of the differentially expressed circRNAs derived from oncogenes and tumor-suppressor genes with homologous mRNAs (Fig. 6B).

**Fig. 7:**
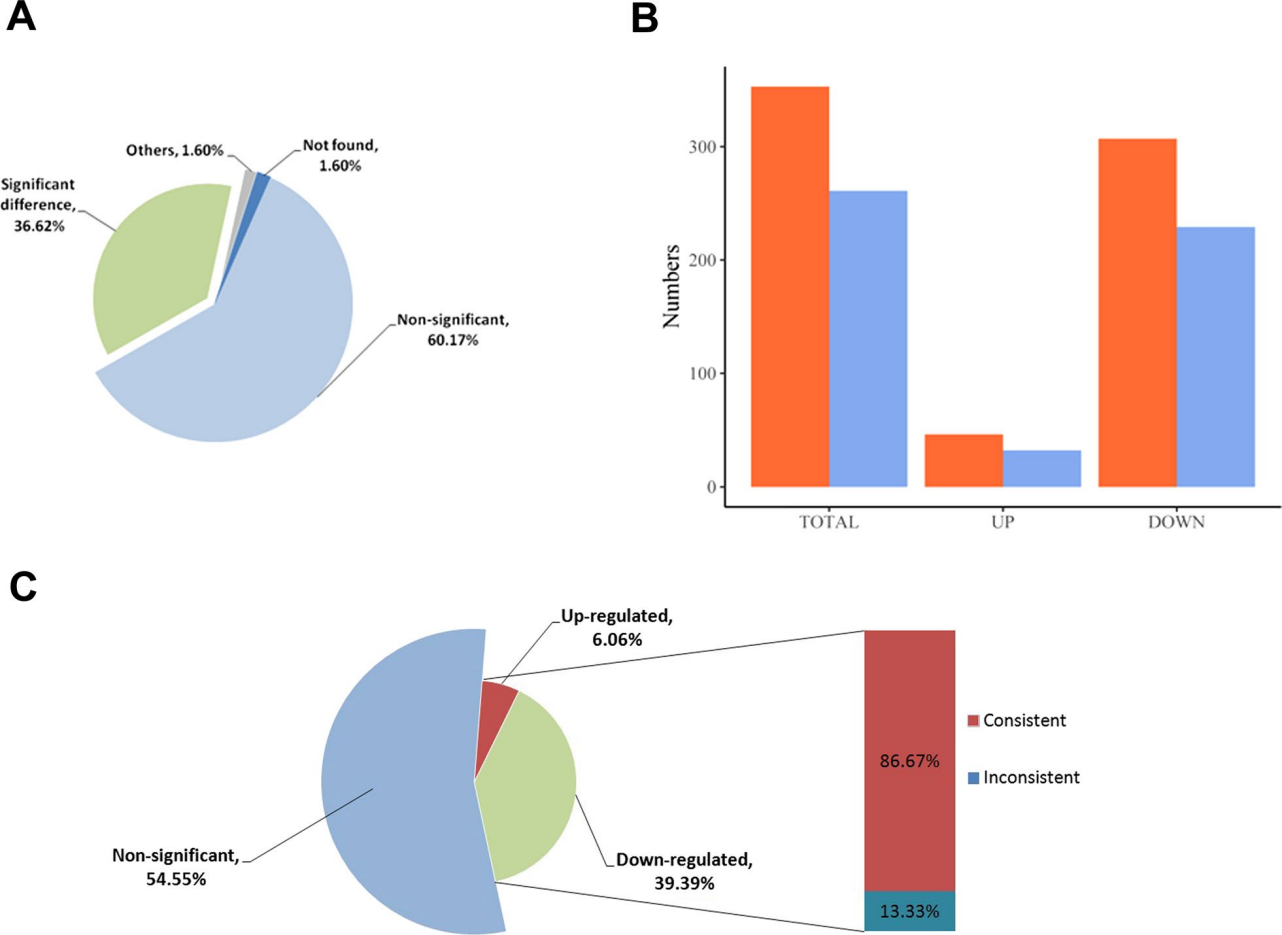
**A** Distribution of miRNA-target genes in tissues. **B** Distribution of miRNA-target circRNAs (blue) in 353 selected circRNAs (orange). **C** Distribution of miRNA-target genes that had relevant circRNAs. The red part of the bar plot showed the proportion of miRNA-target genes consistent with their circular transcripts in expression level

**Fig. 8:**
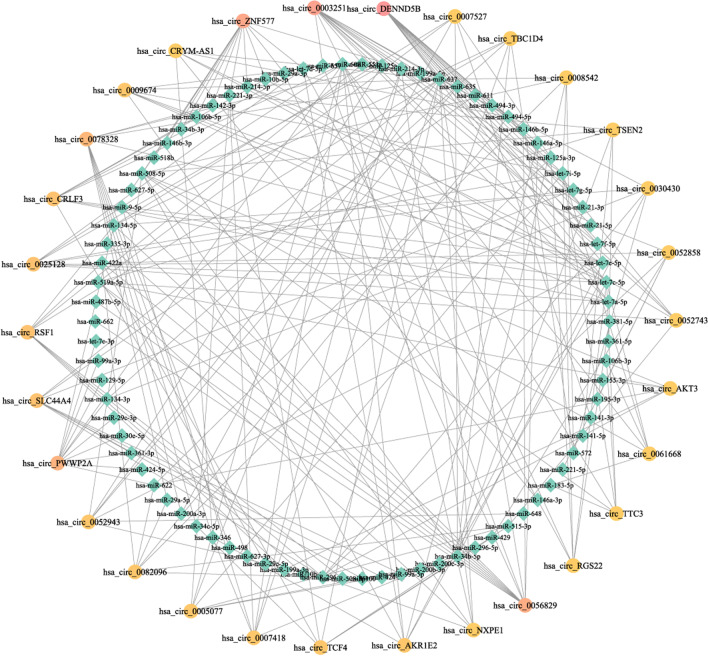
Co-expression network between miRNAs and circRNAs. The rhombic nodes represented miRNAs, and the circular nodes represented circRNAs. The change in circular nodes from yellow to red revealed an increase in the number of targeted miRNAs

To further explore the miRNAs, we introduced some miRNAs that were experimentally confirmed to be related to ovarian cancer from miR2Disease. Mapping these miRNAs to the human MTIs from the miRTarBase revealed 1185 miRNA-target genes validated from reporter assays with strong support. We then found that 98.4% of their linear transcripts appeared in the tissues, and 434 (36.62%) were significantly expressed by characterization of the fold-change (Fig. 7A). Functional analysis classified by variation trend was then conducted, and darker colors of the representative enriched terms corresponded to lower *p*-values (Fig. 3C, D). Pathways linked to cancer were obvious in both groups. For the downregulated groups, biological processes, such as epithelial cell proliferation, vasculature development, and gland development, indicated that they functioned through self-regulation in para-tumor tissues. Enriched terms including signaling by interleukins and signaling by receptor tyrosine kinases reflected their potential as therapeutic targets for ovarian cancer. For example, inhibition of interleukin 6 (IL-6) was found to facilitate therapeutic activity when the anti-IL-6 antibody siltuximab was taken in clinical trials of patients with platinum-resistant ovarian cancer [39]. The receptor-specific tyrosine kinase inhibitor ZD 1839 (‘Iressa’) also contributed to the growth inhibition in ovarian cell lines [40]. For the upregulated group, specific biological processes such as response to wounding, positive regulation of cell migration, negative regulation of cell cycle, and positive regulation of cell death demonstrated the pressure of tumor progression in tissues. Furthermore, 33 miRNA-target genes had relevant circRNAs that were found to be differentially expressed in tissues. A total of 45.45% of these genes were differentially expressed by characterization of the fold-change, and most of them were downregulated in tumor tissues (Fig. 7C). The expression of most of these genes was consistent with the patterns of circRNA expression. Next, the interaction between miRNAs and differentially expressed circRNAs was predicted by miRanda. As mentioned previously, 353 differentially expressed circRNAs (46 upregulated and 307 downregulated) were used for prediction. A total of 261 of them (73.94%) were predicted to target miRNAs related to ovarian cancer, including 32 upregulated and 229 downregulated circRNAs (Fig. 7B). We then calculated the numbers of miRNAs that each circRNAs targeted. Using 16 as the maximum number, circRNAs targeting over 5 miRNAs were used to construct a circRNA-miRNA co-expression network. We used Cytoscape (version 3.6.1) to visualize the complex network (Fig. 8) [41]. In this network, identified circRNAs that were not recorded in circBase were annotated with symbols of their parent genes. A total of 30 circRNAs and 82 disease-related miRNAs (appeared as nodes) were used to establish 235 edges to organize the network, which clearly illustrated their regulatory relationships and the associated pathways.

## Discussion

Ovarian serous cystadenocarcinoma is a type of ovarian epithelial malignancy [2]. Because of the difficulty associated with early diagnosis and treatment, exploring the features at the molecular level is important. Recently, researchers have noticed that the dysregulation of circRNAs might underlie some diseases [11–13]. However, the association between circRNAs and ovarian serous cystadenocarcinoma remains unclear. Here, we explored the expression profile of circRNAs in ovarian serous cystadenocarcinoma.

In this study, we successfully identified many circRNAs in tumor and normal tissues and then conducted an analysis to characterize the differential distribution of these circRNAs. Although the two groups were roughly equal in the number of circRNAs, the distribution of back-spliced reads and quantification demonstrated the marked change in the expression level between the two groups. We detected 353 differentially expressed circRNAs and observed the patterns of expression between tissues. Overall, more circRNAs were weakly expressed in tumor tissues of ovarian serous cystadenocarcinoma. We found that some circRNAs were only significantly expressed in all three specimens of either tumor or normal tissues. These group-specific circRNAs accounted for nearly 59% of the differentially expressed circRNAs. In addition, these circRNAs potentially functioned in tumor progression based on their proportion determined by the analysis with oncogenes and tumor-suppressor genes. Under the stimulation of cancerous process in tissues, the expression of circRNAs involved in tumor progression might change dramatically.

In the enrichment analysis, circRNAs active in the tumor tissues were associated with tumor development, whereas those in the para-tumor tissues were not. We found that 2 of the genes involved in the top 9 clusters in the upregulated group, EPCAM and FGFR2, were oncogenes. Both of these genes were associated with the term R-HSA-1257604: PIP3 activates AKT signaling. This pathway was once thought to be associated with ovarian serous cystadenocarcinoma. Meanwhile, the gene KDM1A, whose products are active in ovarian cancer cells, also appeared in this pathway. Therefore, the pathway R-HSA-1257604: PIP3 activates AKT signaling might be an important pathway in the development of ovarian serous cystadenocarcinoma.

To further explore the function of circRNAs, we attempted to conduct a co-expression analysis with mRNAs and miRNAs. First, we explored the relationship between circRNAs and their parent genes. Given that the production of circRNAs is involved in spliceosomes, circRNAs were thought to compete with their homologous mRNAs for splice sites [15]. The promotion of flanking intronic sequences for circularization might lead to a lower linear splicing efficiency of flanking exons. However, the correlation in this study was the opposite. In addition, this result was obtained based on a screening analysis for the special target genes and their circular isoforms. This consistency was also observed in the analysis with oncogenes and tumor-suppressor genes. For example, circRNAs of the oncogene EPCAM were only expressed in tumor tissues, whereas the expression of relevant mRNAs exhibited a 25-fold increase. In terms of tumor-suppressor genes, MAP2K4 inactivation was confirmed in ovarian cancer cell lines [42]. We also observed clear upregulation of the circRNAs and mRNAs of MAP2K4 in para-tumor tissues. Considering the marked increase in expression level, we hypothesized that competition between transcripts became weaker because of the tumorigenesis in ovarian serous cystadenocarcinoma. Based on the co-expression analysis, our prediction suggested a strong interaction between circRNAs and miRNAs related to ovarian cancer. We noted that miR-608 was targeted by 8 circRNAs upregulated in tumor tissues. miR-608 was thought to inhibit tumor proliferation and migration by targeting special proteins in lung adenocarcinoma and hepatocellular carcinoma [43, 44]. These upregulated circRNAs could thus indirectly contribute to the invasion of ovarian serous cystadenocarcinoma. Additionally, we constructed a complex network to select potential biomarkers in circRNAs. circRNAs can remain stable in cells because their circular structure is more resistant to exonucleolytic activities [45]. This property indicates that circRNAs could be used in clinical diagnosis. Based on the network, half of these circRNAs (15 of 30), which had strong connections with the selected miRNAs, were novel, including hsa_circ_DENND5B. Hsa_circ_DENND5B was found to target 16 miRNAs relevant to ovarian cancer, which was the largest number of miRNAs for a single circRNA. Meanwhile, hsa_circ_0003251 ranked second in the relevant number (14) and was considered a potential miRNA sponge in a previous study examining diabetes and depression [46]. However, hsa_circ_SLC44A4 and hsa_circ_AKR1E2, separately related to 8 and 7 miRNAs, respectively, were noteworthy given their level of enrichment in tumor tissues. miRNAs that hsa_circ_AKR1E2 targeted included miR-608 and 3 miRNAs of the let-7 family. A previous study has suggested that members of the let-7 family can act as tumor suppressors in multiple carcinomas [47]. Therefore, we speculate that these 4 aforementioned circRNAs could be used as potential biomarkers in response to ovarian serous cystadenocarcinoma.

Here, we conducted a multi-angle analysis for circRNAs in ovarian serous cystadenocarcinoma. Several limitations of our study require consideration. The number of specimens was small and did not contain the normal tissues from volunteers free of ovarian serous cystadenocarcinoma. Nevertheless, our study established the expression profile of circRNAs in ovarian serous cystadenocarcinoma and provides a foundation for future studies to validate these findings.

## Conclusions

In this study, we characterized the expression profile of circRNAs in ovarian serous cystadenocarcinoma and conducted a co-expression analysis with mRNAs and miRNAs. We identified 15,092 unique circRNAs and clarified their patterns of differential expression in tissues. Enrichment analysis and prediction of the interactions with 353 differentially expressed circRNAs indicated that the function of circRNAs was to facilitate tumor progression, suggesting the presence of a potential pathway in ovarian serous cystadenocarcinoma. Moreover, consistent changes between circRNAs and the homologous mRNAs might be associated with latent regulation in ovarian serous cystadenocarcinoma. After network analysis, several circRNAs were determined to represent possible biomarkers. Generally, our study established the role of circRNAs in the tumorigenesis of ovarian serous cystadenocarcinoma and provides a basis for future work.

## Supplementary Information

**Additional file 1: Fig. S1**. The sequence depths of six specimens.

## Data Availability

The sequencing data have been deposited in the GSA-Human datasets of the National Genomics Data Center in China (https://bigd.big.ac.cn/) under the accession number PRJCA002938.

## Abbreviations

circRNAs: Circular RNAs
miRNA: MicroRNA
GO: Gene ontology
pre-mRNA: Precursor mRNA
polyA: Polyadenylated
MTIs: MiRNA–target interactions
RPM: Reads per million mapped reads
pAKT: Phosphorylated AKT
