# Supplementary figures and images for: The circular RNA expression profile in ovarian serous cystadenocarcinoma reveals a complex circRNA–miRNA regulatory network
Source: BMC Med Genomics. 2021 Dec 2;14(Suppl 2):276. doi: 10.1186/s12920-021-01132-5 (PMC8638095; doi:10.1186/s12920-021-01132-5)

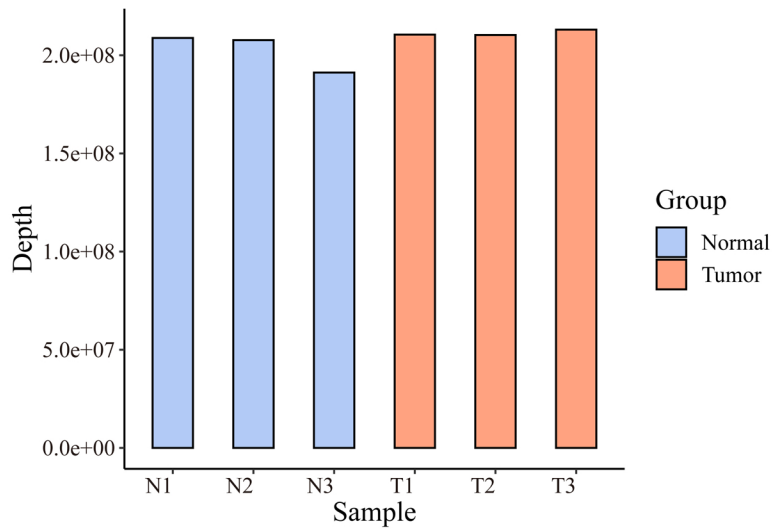

| Sample      | N1        | N2        | N3        | T1        | T2        | T3        |
|-------------|-----------|-----------|-----------|-----------|-----------|-----------|
| No.of reads | 208808109 | 207694179 | 191195088 | 210529069 | 210322748 | 213062070 |

Supplement: Supplementary file 1 — Additional file 1: Fig. S1. The sequence depths of six specimens. [file 12920_2021_1132_MOESM1_ESM.pdf]
